# Melittin Constrains the Expression of Identified Key Genes Associated with Bladder Cancer

**DOI:** 10.1155/2018/5038172

**Published:** 2018-05-03

**Authors:** Zidan Jin, Jie Yao, Nianlin Xie, Libo Cai, Shuai Qi, Zhan Zhang, Bai Li

**Affiliations:** ^1^Department of Rehabilitation Medicine, Changhai Hospital, Second Military Medical University, Shanghai 200433, China; ^2^Department of Urological Surgery, The 161th Hospital of PLA, Wuhan, Hubei Province 430012, China; ^3^Department of Urological Surgery, Zhongnan Hospital of Wuhan University, Wuhan, Hubei Province 430071, China; ^4^Department of Thoracic Surgery, Tangdu Hospital, Fourth Military Medical University, Xi'an, Shanxi Province 710038, China; ^5^Department of Accident and Emergency, Heihe No.1 People's Hospital, Heihe 164300, China; ^6^Department of Pharmacy, The 161th Hospital of PLA, Wuhan, Hubei Province 430012, China

## Abstract

This work is aimed at investigating the effect of melittin on identified key genes in bladder cancer (BC) and further providing a theoretical basis for BC treatment. GSE35014 downloaded from the Gene Expression Omnibus (GEO) database was used to screen differentially expressed genes (DEGs) in BC cells and control. Results showed that a total of 389 upregulated and 169 downregulated genes were identified. Subsequently, GO analysis, KEGG pathway enrichment analysis, and PPI network analysis were employed to disclose the crucial genes and signaling pathways involved in BC. Fifteen module-related DEGs and their associated signaling pathways were obtained according to the PPI network and modular analyses. Based on the analysis of articles retrieved in the PubMed database, we found that melittin could induce apoptosis and constrain the progression of tumor cells as a result of regulating critical cancer-related signaling pathways, such as PI3K-Akt and TNF signaling pathways. Furthermore, PI3K-Akt and TNF signaling pathways were also found to be associated with module-related DEGs according to biological analyses. At last, qRT-PCR analysis demonstrated that melittin could constrain the expression of module-related DEGs (LPAR1, COL5A1, COL6A2, CXCL1, CXCL2, and CXCL3) associated with PI3K-Akt and TNF signaling pathways in BC cells. Functional assays revealed that melittin could constrain the proliferative and migrated abilities of BC cells. Conjointly, these findings provide a theoretical basis for these six genes as drug-sensitive markers of melittin in BC treatment.

## 1. Introduction

Commonly accepted, bladder cancer (BC) pertains to a malignant tumor with top incidence in urological diseases [1]. Overall, bladder tumors are commonly in the presence of noninvasive urothelial carcinoma and muscle-invasive disease [2]. The invasive subtype of BC is closely associated with metastatic spread and poor prognosis of patients [3]. Despite great advances made in the diagnosis and treatment of BC, therapeutic options are limited and prognosis remains unfavorable [4, 5]. Therefore, elucidating the underlying molecular mechanism of development and progression is conductive to and even decisive on BC treatment.

The application of Chinese traditional medicine in tumor treatment has gained much attention in recent years [6–8]. Melittin with twenty-six amino acids is the essential component of honeybee venom [9]. Prior research generally confirms that honeybee venom is accepted as Chinese medicine and applied for treatment of various diseases, exemplified by arthritis, rheumatism, back pain, cancer, and skin disease [10]. Intriguingly, discussions regarding melittin associated with diverse cancers have dominated research in recent years. In our previous work, we retrieved and analyzed articles from 2010 to now in the PubMed database with the search keyword of melittin and cancer. We found that melittin could induce apoptosis and constrain the progression of tumor cells as a result of regulating critical cancer-related signaling pathways. For example, Jo et al. unveiled that melittin could have an anticancer effect as a result of inducing apoptotic cell death in ovarian cancer through enhancement of death receptors and suppression of the JAK2/STAT3 signaling pathway [11]. A similar anticancer effect in prostate cancer cells was evidenced and materialized through activation of the caspase pathway via inactivation of NF-*κ*B [12]. It has also been reported that melittin could suppress PMA-induced tumor cell invasion by inhibiting NF-*κ*B- and AP-1-dependent MMP-9 expression [13], probably through JNK/p38- and NF-*κ*B-dependent mechanisms [14]. Jeong et al. outlined that melittin suppresses TNF-*α*-induced HASMC migration through the selective inhibition of MMP-9 expression [15]. Shin et al. illustrated that melittin could hold down HIF-1*α*/VEGF expression through inhibition of the ERK and mTOR/p70S6K pathway in human cervical carcinoma cells [16]. Jeong et al. probed that melittin could constrain EGF-induced MMP-9 expression via blocking the NF-*κ*B and PI3K/Akt/mTOR signaling pathway and repress EGF-induced FAK phosphorylation through inhibiting the mTOR/p/0S6K/4E-BP1 signaling pathway in breast cancer cells [17]. Kong et al. proffered the conclusion of melittin exerting a positive role in gastric cancer cell apoptosis via activation of the mitochondrial pathway [18]. Furthermore, the antitumor activity of melittin was reported to be associated with antiangiogenic actions via inhibiting VEGFR-2 and inflammatory mediators involved in the MAPK signaling pathway [19] or to suppress hepatic epithelial-to-mesenchymal transition via blocking of TGF*β*/Smad and MAPK-JNK signaling pathways [20]. As recently reported, it was demonstrated that melittin could constrain tumor angiogenesis modulated by endothelial progenitor cells involved in the SDF-1*α*/CXCR4 signaling pathway in a UMR-106 osteosarcoma xenograft mouse model [21]. However, little was known about the association between melittin and BC, which promoted us to conduct this study.

Popularly accepted, the Gene Expression Omnibus (GEO) accessible at www.ncbi.nlm.gov/geo is a public repository that archives and freely distributes high-throughput gene expression data submitted by the scientific community. Huge volumes of data including individual gene expression, multifarious organisms, and a wide range of biological issues could be effectively explored, queried, and visualized by means of user-friendly web-based tools. In recent years, microarrays contingent on high-throughput platforms have emerged as a promising and efficient tool for screening significant genetic or epigenetic alternations in carcinogenesis and identifying promising biomarkers for diagnosis and prognosis of cancers [22]. Plentiful gene expression profiling microarrays have been employed to identify multifarious differentially expressed genes (DEGs) in various cancers, and a number of BC-related DEGs have therefore been documented [23–25]. Nonetheless, various additional critical genes and pathways associated with BC remain yet to be explored completely.

**Figure 1:**
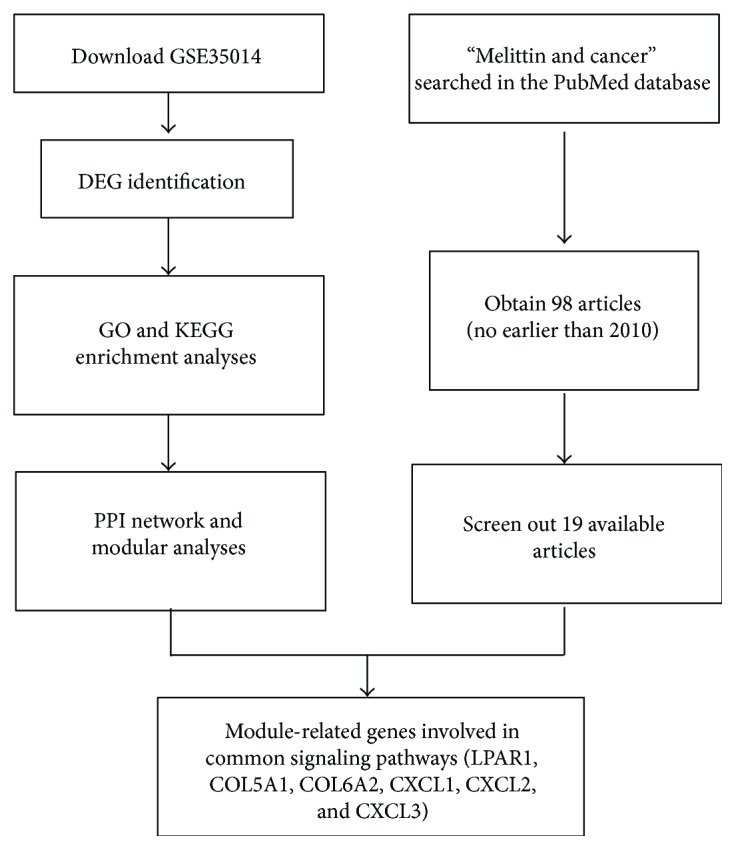
Data analysis pipeline for key genes in BC. DEGs: differentially expressed genes; GO: gene ontology; KEGG: Kyoto Encyclopedia of Genes and Genomes; PPI: protein–protein interaction; BC: bladder cancer.

In the present study, we aimed to disclose the crucial genes associated with BC and investigate the effect of melittin on the identified crucial genes in BC. On the one hand, the transcription profile of GSE35014 was downloaded from the GEO database (https://www.ncbi.nlm.nih.gov/geo/query/acc.cgi?acc=GSE35014) and data processing was performed using R software (version 3.3.0, available online: https://www.r-project.org/) and bioconductor packages (available online: http://www.bioconductor.org/), together with the online website Database for Annotation, Visualization, and Integrated Discovery (DAVID; version 6.8). Differentially expressed genes (DEGs) were screened in BC cells and control. Gene ontology (GO) analysis, Kyoto Encyclopedia of Genes and Genomes (KEGG) pathway enrichment analysis, and protein–protein interaction (PPI) network analysis were employed to evaluate the crucial genes and signaling pathways involved in BC. On the other hand, we collected melittin-related articles involved in cancers from the PubMed database and analyzed the relationship between melittin and various cancers that have been reported. After that, we combined the KEGG, PPI network, and module analyses intersecting with the literature analysis to obtain our target signaling pathway and crucial DEGs. The data analysis pipeline for key genes in BC is displayed in Figure 1. Finally, we investigated the effect of melittin on the critical DEG expression in BC cells and its functional role in proliferative and migrated abilities of BC separately by qRT-PCR and functional assays. By this token, these critical DEGs and signaling pathways identified in this work would convey valuable information about the mechanism of BC carcinogenesis and expose the discovery of key genes that may act as future targets of anticancer therapy.

## 2. Materials and Methods

### 2.1. Dataset Collection and Identification of DEGs in BC

Microarray technology becomes widely accepted as a high-throughput tool for measuring gene expression [26]. As previously reported, data from DNA microarray analysis plays a reliable and useful role in identifying novel targets for clinical diagnostic and therapeutic approaches [27, 28]. The selection criteria of datasets in our work were described as follows: (1) associated with BC cells, (2) focus on the invasion and metastasis of BC, (3) sample size no less than 4, and (4) based on the GPL570 platform. Extracted from the GEO (https://www.ncbi.nlm.nih.gov/geo/) database, one microarray expression profile termed GSE35014 was therefore employed to identify DEGs associated with BC (https://www.ncbi.nlm.nih.gov/geo/query/acc.cgi?acc=GSE35014). The microarray data of GSE35014 was contingent on GPL570 platforms (Affymetrix Human Genome U133 Plus 2.0 Array, Affymetrix Inc., Santa Clara, CA, USA). It has been reported that UM-UC-3 bladder carcinoma cells have lost the endogenous expression of Rho GDP dissociation inhibitor 2 (RhoGDI2), as occurs commonly in the progression of BC [29]. In this query, two paired biological replicates of GFP (control) and GFP-RhoGDI2 (experimental) UM-UC-3 cells were constructed at different times, isolated during log phase growth, and subjected to gene expression profiling by means of the Affymetrix HG-U133 Plus 2.0 oligonucleotide microarray platform.

R software (version 3.3.0; https://www.r-project.org/) and bioconductor packages (available online: http://www.bioconductor.org/) were applied for data mining and statistical analyses. Concretely, the original annotation file and CEL files of the Affymetrix platform were firstly downloaded. Limma package [30] was applied to identify DEGs between experimental and control groups with the cut-off criterion of *P* < 0.05 and absolute log2 FC > 1.

### 2.2. GO and KEGG Enrichment Analyses in BC

With regard to the investigation of potential biological functions of DEGs in BC, GO [31] functional and KEGG [32] pathway analyses were conjointly carried out. The DAVID online tool available at https://david.ncifcrf.gov/ was applied for these analyses. Significantly enriched GO terms and KEGG pathways were screened out as a criteria of *P* < 0.05, finally visualized using R package ggplot2 [33].

### 2.3. PPI Network Construction

Search Tool for the Retrieval of Interacting Genes (STRING) accessible at http://string-db.org/ is an online database which includes experimental data and computational prediction methods as well as public text collections, involved in beyond 1100 completely sequenced organisms [34]. The protein interactions in the STRING database were presented as a combined score. The STRING database system was used to construct the functional protein association networks. Interacting pairs with a combined score more than 0.8 were considered to be significant and employed to construct networks. Cytoscape (http://www.cytoscape.org/) [35] was disseminated to visualize these networks.

### 2.4. MCODE Analysis

Molecular Complex Detection (MCODE; https://omictools.com/molecular-complex-detection-tool) could be applied to evaluate densely connected regions in large PPI networks possibly involved in molecular complexes [36]. MCODE was disseminated to mine the core protein complex in the PPI network constructed in this work.

### 2.5. Cell Culture and Treatment

The human BC cell lines T24 and 5637 were purchased from the American Type Culture Collection (ATCC; Manassas, VA). Cells were cultured in Dulbecco's modified Eagle's medium (DMEM; Gibco BRL, Grand Island, NY, USA) supplemented with 10% fetal bovine serum (FBS; Gibco BRL, Grand Island, NY, USA) and 100 U/ml of penicillin/streptomycin. The cultured cells were maintained at 37°C in a humidified atmosphere with 5% (*v*/*v*) CO_2_. Cells were plated in 24-well plates (5 × 10^4^ cells/well), and subconfluent cells were treated with melittin at a concentration of 0 and 4 *μ*g/ml for 24 h, respectively. Melittin used in this work was purchased from Sigma-Aldrich (St. Louis, MO, USA).

### 2.6. Real-Time Quantitative PCR Validation

Total RNA was extracted from treated cells using the TRIzol reagent (Life Technologies, California, USA) according to the manufacturer's instructions. 1 *μ*g of the total RNA was withdrawn and used as the template for cDNA synthesis using a PrimeScript RT Reagent Kit with cDNA Eraser (Takara Biotech, Dalian, China). Quantitative real-time polymerase chain reaction (qRT-PCR) was performed by using SYBR Premix Ex Taq (Takara Biotech, Japan) on an ABI 7900 system (Applied Biosystems, Foster City, CA, USA). The primers used in this work are presented in Table 1. The housekeeping gene glyceraldehyde 3-phosphate dehydrogenase (GAPDH) was used as an internal control. The quantification of gene expression was calculated using a relative quantification method (2^−ΔΔCt^).

### 2.7. Cell Proliferation Assays

After treatment with melittin (0 and 4 *μ*g/ml), cell proliferation was assessed by the MTS assay (Promega, Madison, US) according to the manufacturer's protocol. 5637 and T24 cells (2000 cells per well) in each group were plated in 96-well plates. Twenty microliters of the MTS reagent was added to each well containing 100 *μ*l of culture medium. The plate was incubated for 2 h at 37°C in a humidified atmosphere with 5% (*v*/*v*) CO_2_. The plate was then read at 490 nm using a plate reader.

### 2.8. Cell Migration Assays

The scratch wound-healing assay was employed to examine cell migration. Uniform wounds were scraped in 5637 and T24 cells grown on plastic 6-well plates using a pipette tip before transfection. The initial gap length (0 h) and the residual gap length (48 h) after wounding were calculated from photomicrographs.

### 2.9. Statistical Analysis

Each experiment was repeated three times, and all values were presented as the means ± standard deviation (SD). The *t*-test was employed to analyze the difference in two groups. GraphPad Prism software version 5.0 was applied to complete statistical analysis. A value of *P* < 0.05 was considered statistically significant in this work.

## 3. Results

### 3.1. Identification of DEGs in BC

**Figure 2:**
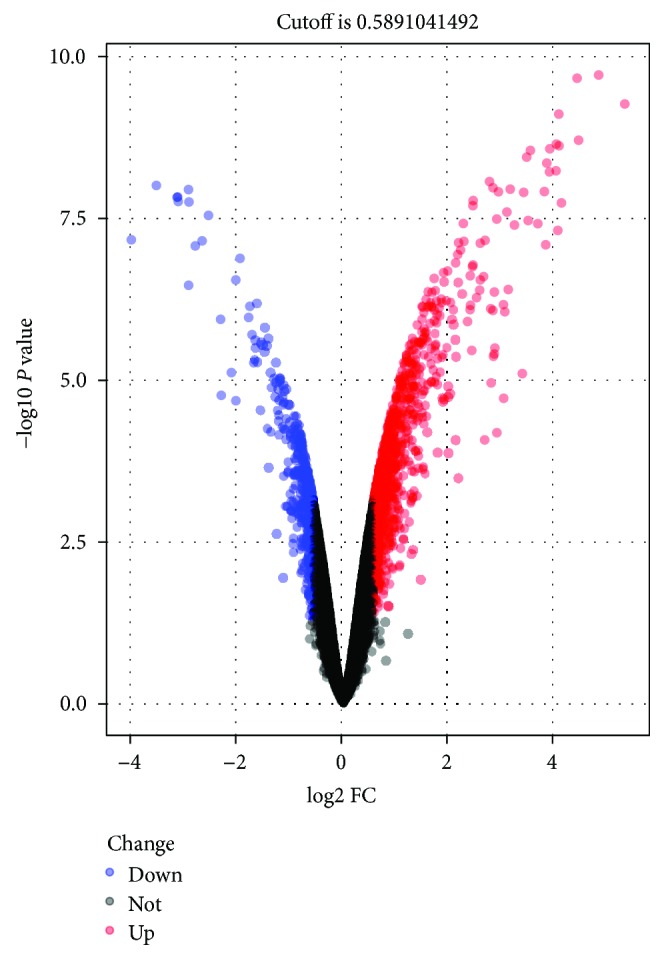
Volcano plot of detectable DEG profiles in BC cells. Red plots stand for upregulated genes, and blue ones represent downregulated genes with *P* < 0.05 and absolute log_2_FC > 1. Black ones indicate those nonsignificant expressed genes. The abscissa means the value of fold change in circulating the gene expression between BC cells and control. The ordinate shows the −log_10_ of the adjusted *P* value for each gene, suggesting the strength of the association. DEGs: differentially expressed genes; BC: bladder cancer; FC: fold change.

According to microarray analysis, a total of 558 DEGs were achieved to be differentially expressed with a fold change > 2 between the BC cell group and control, as revealed in Figure 2. Among them, 389 upregulated and 169 downregulated genes were separately obtained as listed in Table 2. These DEGs were employed for follow-up analyses.

### 3.2. GO Enrichment Analysis

**Figure 3:**
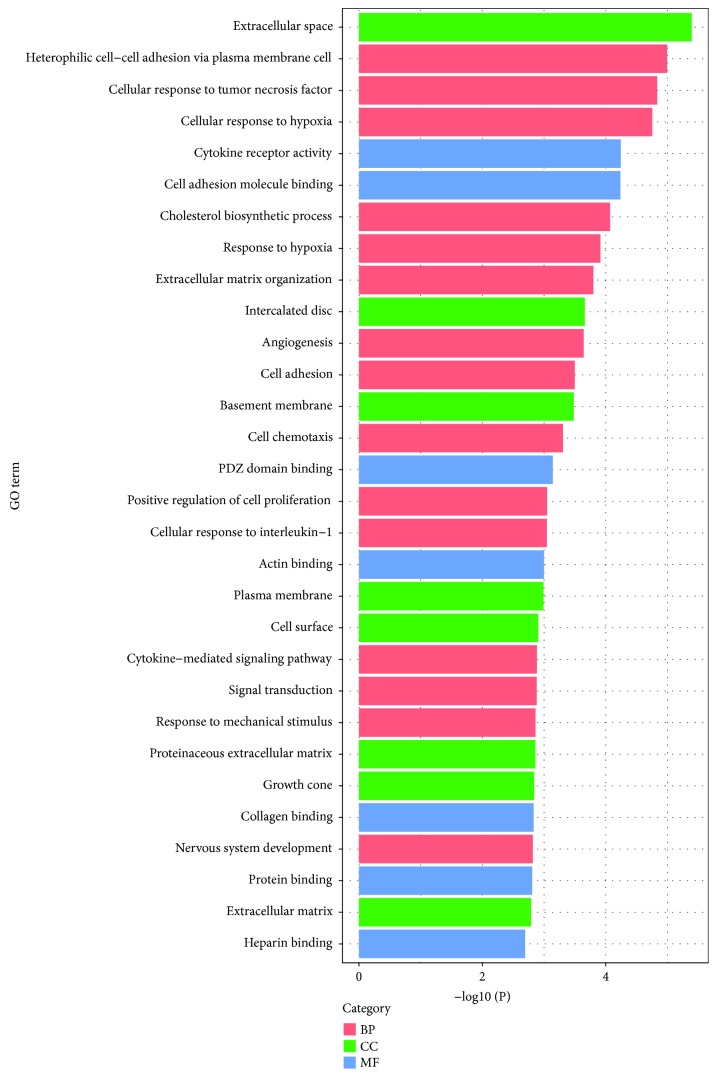
Top thirty significant enriched GO terms of DEGs in BC according to their functions. GO: gene ontology; BP: biological process; MF: molecular function; CC: cellular component; DEGs: differentially expressed genes; BC: bladder cancer.

Commonly, GO functional enrichment analysis is involved in biological process, molecular function, and cellular component. In this study, GO functional enrichment analysis of DEGs was performed by means of an online biological tool DAVID with a threshold of *P* < 0.05. Top thirty significant GO terms according to *P* value are visualized and displayed in Figure 3, and corresponding related DEGs are summarized in Table 3. It could be materialized that a sum of DEG-enriched terms play a critical role in the biological behavior of tumor cells. Most of the DEGs were significantly enriched in protein binding, plasma membrane, extracellular space, and signal transduction. Concretely, in terms of biological process, DEGs were principally enriched in signal transduction, cell adhesion, and positive regulation of cells, while they are concentrated in the plasma membrane, extracellular space, and cell surface with respect to cellular component. As for molecular function, DEGs focused on the relation with protein binding, actin binding, and heparin binding.

### 3.3. Signaling Pathway Enrichment Analysis

**Figure 4:**
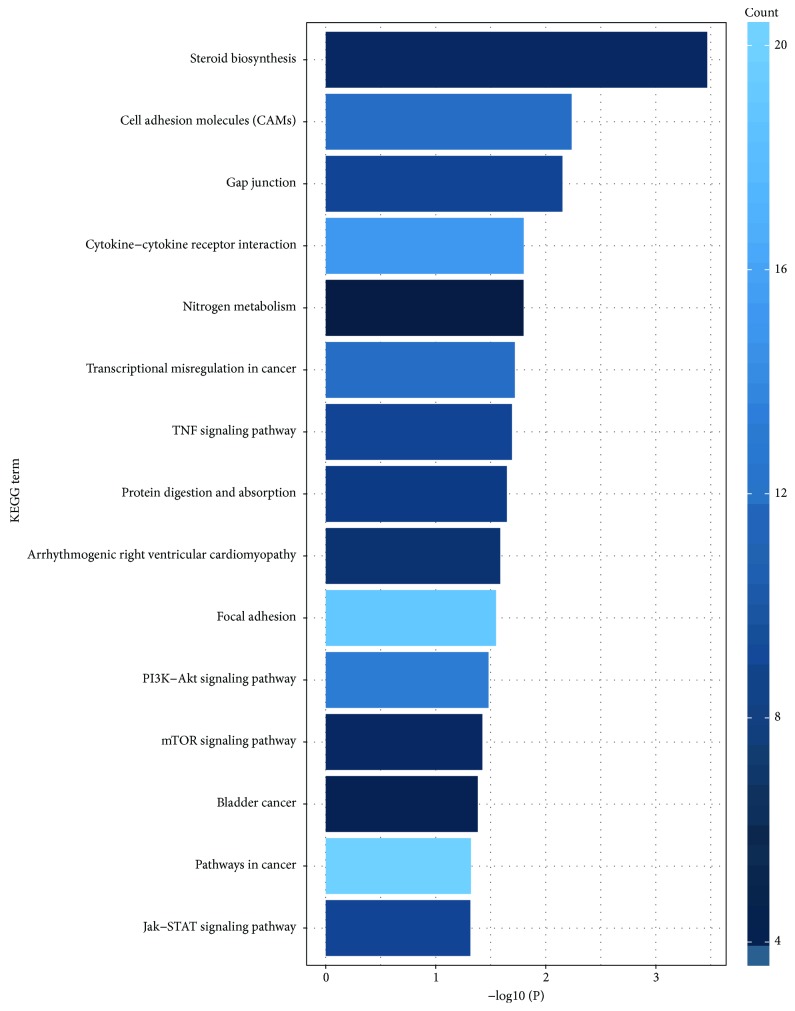
Top fifteen significant pathways associated with the DEGs according to *P* value in KEGG analysis. KEGG: Kyoto Encyclopedia of Genes and Genomes; DEGs: differentially expressed genes.

Pathway analyses were used to identify the significant pathways associated with the DEGs according to KEGG analysis. As mentioned in GO analysis, the online biological tool DAVID with a threshold of *P* < 0.05 was also applied in this section. Top fifteen significant pathways were visualized by R package ggplot2. As depicted in Figure 4 and Table 4, DEGs were essentially abundant in cancer-related pathways, such as pathways in cancer (ID: hsa05200), PI3K-Akt signal pathway (ID: hsa04151), cytokine-cytokine receptor interaction (ID: hsa04060), focal adhesion (ID: hsa04510), cell adhesion molecules (ID: hsa04514), transcriptional misregulation in cancer (hsa05202), Jak-STAT signal pathway (ID: hsa04630), and TNF signaling pathway (ID: hsa04668), which all play a critical and even decisive role in the progression and development of tumors.

### 3.4. Identification of Key Candidate Genes and Signaling Pathways Based on PPI Network and Modular Analyses

**Figure 5:**
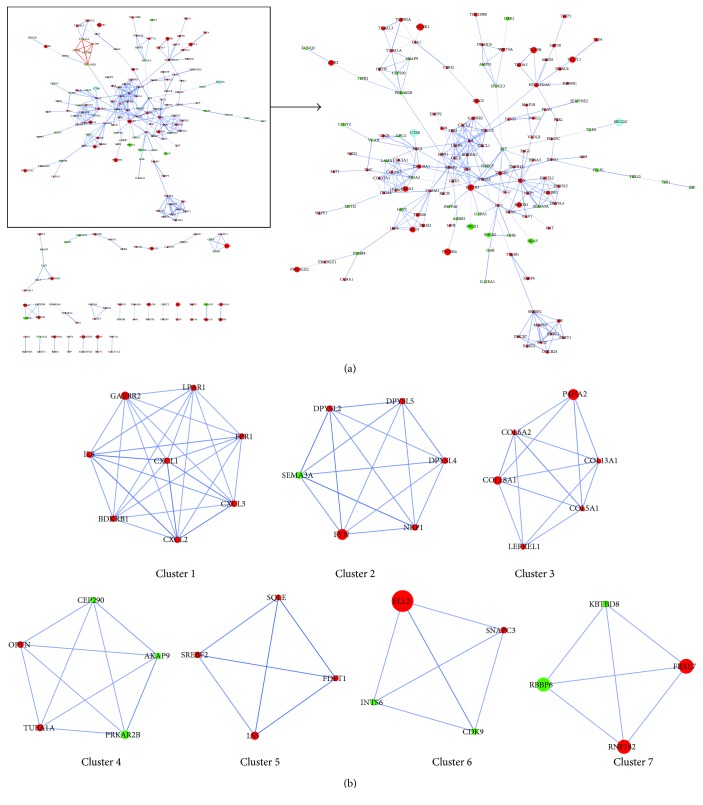
PPI network constructed by Cytoscape and top seven module networks by MCODE. (a) The PPI network was constructed dependent on the STRING online database (available online: http://string-db.org) and Cytoscape software (version 3.4.0, available online: http://www.cytoscape.org/). (b) The top seven module networks with a score no less than 4 were analyzed by MCODE. The red nodes stand for upregulated genes, and the green nodes stand for downregulated genes. The larger the node diameter is, the smaller the *P* value and the more significant the node is. PPI: protein–protein interaction; MCODE: Molecular Complex Detection; STRING: Search Tool for the Retrieval of Interacting Genes.

With the purpose of systemically analyzing the functions of DEGs in BC, these DEGs were mapped to PPI data dependent on the STRING online database (available online: http://string-db.org) and Cytoscape software (version 3.4.0, available online: http://www.cytoscape.org/). Interacting pairs with a combined score more than 0.8 were considered to be significant and employed to construct networks. The PPI network with 309 interacting pairs was thus obtained (Figure 5(a)), which also was provided as Supplementary Material (available here) for zooming in and out as needed. Based on the PPI network, we subsequently analyzed the network clusters by MCODE, and twelve module networks were therefore generated as described in Table 5. The module networks with a score no less than 4 were selected for the next analysis and are depicted in Figure 5(b). Fifteen module-related DEGs marked as bold in Table 4 were thus obtained, including CXCL2, CXCL1, BDKRB1, LPAR1, CXCL3, FYN, COL6A2, COL18A1, COL13A1, COL5A1, TUBA1A, FDFT1, SQLE, LSS, and CDK9. Combined with Tables 4 and 5, it could be found that the signaling pathways associated with these module-related DEGs were described as follows: pathways in cancer (ID: hsa05200), PI3K-Akt signaling pathway (ID: hsa04151), focal adhesion (ID: hsa04510), transcriptional misregulation in cancer (ID: hsa05202), TNF signaling pathway (ID: hsa04668), Gap junction (ID: hsa04540), protein digestion and absorption (ID: hsa04974), and steroid biosynthesis (ID: hsa00100).

### 3.5. Melittin Constrained the Expression of the Module-Related DEGs Associated with PI3K-Akt and TNF Signaling Pathways in BC Cells

**Figure 6:**
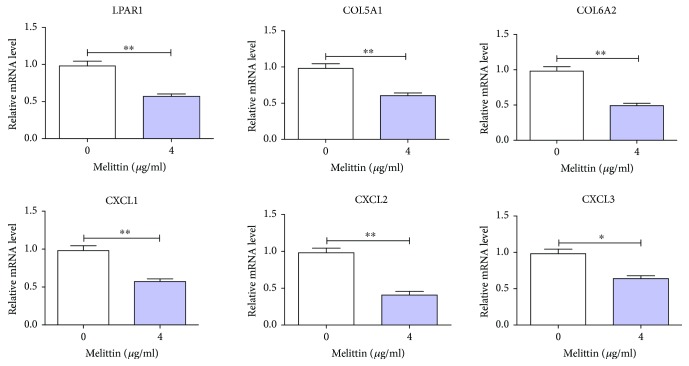
The expressions of the module-related DEGs associated with PI3K-Akt and TNF signaling pathways were detected in T24 cells treated with melittin by qRT-PCR. DEGs: differentially expressed genes. ^∗^ *P* < 0.05 and ^∗∗^ *P* < 0.01.

According to literature analysis, we found that the signaling pathways associated with melittin in cancers mainly include JAK2/STAT3, NF-*κ*B, JNK/p38/NF-*κ*B, TNF-*α*, mTOR/p70S6K or mTOR/p/0S6K/4E-BP1, PI3K/Akt/mTOR, mitochondrial pathway, MAPK signaling pathway, TGF*β*/Smad and MAPK-JNK signaling pathways, and SDF-1*α*/CXCR4 signaling pathway so far [11–21]. JAK2/STAT3, TNF-*α*, mTOR/p70S6K or mTOR/p/0S6K/4E-BP1, and PI3K/Akt/mTOR signaling pathways represented above were in accordance with the signaling pathway IDs of hsa04630, hsa04668, hsa04150, and hsa04151 as shown in Table 4. The module-related DEGs in the PI3K/Akt (namely, LPAR1, COL5A1, and COL6A2) and TNF signaling pathway (termly CXCL1, CXCL2, and CXCL3) were selected to investigate the effect of melittin on them in BC cells. As revealed in Figure 6, it was clear that the expressions of LPAR1, COL5A1, and COL6A2 associated with the PI3K/Akt signaling pathway were significantly decreased in cells treated with melittin compared to the control. So was the case in the expressions of CXCL1, CXCL2, and CXCL3 involved in the TNF signaling pathway. Collectively, our findings demonstrated that melittin could constrain the expression of the module-related DEGs associated with PI3K-Akt and TNF signaling pathways in BC cells.

### 3.6. Melittin Suppressed Cell Proliferation and Migration in BC Cell Lines

**Figure 7:**
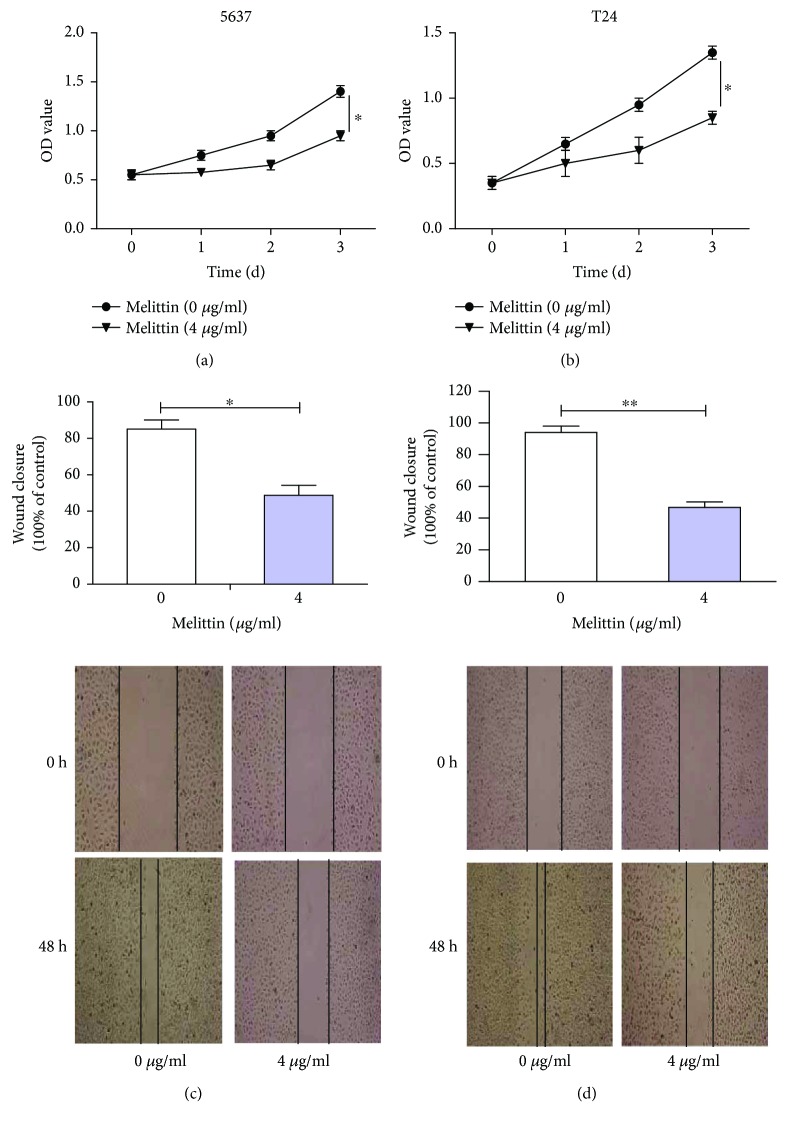
Melittin suppressed cell proliferation and migration in 5637 and T24 cell lines. (a) 5637 and (b) T24 cell viabilities were significantly inhibited in the melittin-treated group (4 *μ*g/ml) in comparison with the control group (0 *μ*g/ml) as determined by the MTS assay. (c) 5637 and (d) T24 cell migration was remarkably constrained in the melittin-treated group (4 *μ*g/ml) in comparison with the control group (0 *μ*g/ml) as analyzed by the scratch wound-healing assay. Magnification ×100. OD: optical density. ^∗^ *P* < 0.05 and ^∗∗^ *P* < 0.01.

As previously reported, melittin could have an inhibitory effect on cell motility and migration through PI3K/Akt and TNF signaling pathways in diseases [15, 17]. We therefore investigated the effect of melittin on cell proliferation and migration in 5637 and T24 cell lines by MTS and scratch wound-healing assays. Results obtained showed that cell viabilities were significantly inhibited in the melittin-treated group (4 *μ*g/ml) in comparison with the control group (0 *μ*g/ml) according to the MTS assay (Figures 7(a) and 7(b), *P* < 0.05). Similar results were also obtained in the effect of melittin on cell migration as analyzed by the scratch wound-healing assay (Figures 7(c) and 7(d), *P* < 0.05). Taken together, melittin could induce a significant reduction in proliferation and migration of BC cells.

## 4. Discussion

As we know, melittin with twenty-six amino acids is the essential component of honeybee venom [9]. Current available literature including in vitro and in vivo studies suggests that melittin governs signal transduction and regulatory pathways associated with multiple cancer death mechanisms, including inhibition of proliferation, induction of apoptosis, inhibition of angiogenesis, cell cycle arrest, and inhibition of cancer motility, migration, metastasis, and invasion [37]. The melittin-related cancers reported consist of leukemia [38], lung carcinoma mouse model [39], pancreatic ductal adenocarcinoma [40], hepatocellular carcinoma cells [41], ovarian cancer cells [11], and so forth. However, little was known about the association between melittin and BC. Melittin and bladder cancer were set as search keywords in the PubMed database, and only three related articles were obtained [42–44]. It was worth noting that the associated signaling pathways involved in melittin regulating BC also remained yet to be completely elucidated.

In the present work, a genome-wide survey and literature search were therefore conjunctively employed to identify the potential key genes and signaling pathways associated with melittin in BC. 389 upregulated and 169 downregulated DEG genes were obtained according to dataset analysis contingent on high-throughput platforms, enriching the document of BC-related DEGs now available. GO [31] functional enrichment analysis evidenced that most of the DEGs were significantly enriched in protein binding, plasma membrane, extracellular space, and signal transduction. KEGG [32] analysis surfaced that DEGs were essentially abundant in pathways in cancer, PI3K-Akt signal pathway, Jak-STAT signal pathway, TNF signaling pathway, and mTOR signaling pathway. We noted that JAK2/STAT3 [11], TNF-*α* [15], mTOR/p70S6K or mTOR/p/0S6K/4E-BP1 [16], and PI3K/Akt/mTOR [17] signaling pathways associated with melittin in cancers were in accordance with the signaling pathway IDs of hsa04630, hsa04668, hsa04150, and hsa04151. Ulteriorly, fifteen module-related DEGs were obtained according to PPI network and modular analyses with a score no less than 4, namely, CXCL2, CXCL1, BDKRB1, LPAR1, CXCL3, FYN, COL6A2, COL18A1, COL13A1, COL5A1, TUBA1A, FDFT1, SQLE, LSS, and CDK9. Combining KEGG with module analysis, the module-related DEGs corresponding to signaling pathways were described as follows: SQLE, LSS, and FDFT1 for steroid biosynthesis (ID: hsa00100); LPAR1 and TUBA1A for gap junction (ID: hsa04540); CDK9 for transcriptional misregulation in cancer (ID: hsa05202); CXCL1, CXCL3, and CXCL2 for the TNF signaling pathway (ID: hsa04668); COL18A1, COL13A1, COL6A2, and COL5A1 for protein digestion and absorption (ID: hsa04974); LPAR1, COL5A1, and COL6A2 for the PI3K-Akt signaling pathway (ID: hsa04151); COL5A1, FYN, and COL6A2 for focal adhesion (hsa04510); and BDKRB1 and LPAR1 for pathways in cancer (ID: hsa05200). It was clear that the PI3K-Akt (ID: hsa04151) and TNF signaling pathways (ID: hsa04668) associated with melittin contained the module-related DEGs in combination of literature and bioinformatic analyses. The module-related DEGs contained in these two signaling pathways were thus selected for further work. The effect of melittin on their contained module-related DEGs, LPAR1, COL5A1, COL6A2, CXCL1, CXCL2, and CXCL3, was subsequently analyzed in BC cells. In fact, some of these module-related DEGs have been reported to be significantly correlated with the occurrence and development of BC, which were exemplified as follows. Zhang et al. reported that the CXCL2-associated signaling pathway, CXCL2/MIF-CXCR2, could advance the recruitment of myeloid-derived suppressor cells and be identified as predictors and potential therapeutic targets in BC patients [45]. CXCL1 as a potential mediator and marker of the tumor invasion of BC was elevated in the urine of BC patients [46, 47]. CXCL1 and CXCL3 related to the nuclear factor kappa B and nuclear factor (erythroid-derived 2)-like 2 signaling pathways were measured in human urothelial carcinoma cell lines [48]. Expression changes of COL3A1 and COL5A1 as the fibrillar collagen proteins were associated with muscle-invasive bladder transition cell carcinoma [49]. Our results showed that these module-related genes were downregulated in cells treated with melittin compared to the control, indicating that melittin could constrain the expression of the module-related DEGs associated with PI3K-Akt and TNF signaling pathways in BC. This further highlights that these genes might be drug-sensitive markers for melittin in BC treatment. As previously reported, melittin exerts an antitumor effect on non-small-cell lung cancer cells [50] and suppresses EGF-induced cell motility and invasion in breast cancer cells [17]. In our work, the functional assays conveyed the prohibitive role of melittin in proliferative and migrated abilities of BC cell lines. This may be the first evidence regarding the initiatory role of melittin in BC metastasis.

To conclude, our findings conveyed valuable information about the mechanism of BC carcinogenesis and expose the discovery of key genes that may act as future targets of anticancer therapy. However, only preliminary verification was conducted regarding the effect of melittin on the expression of the key genes in BC cells in this work, and further investigation remains yet to be elucidated. Therefore, the functional role and concrete mechanism of melittin on the crucial genes in BC would move forward in our next plan on the basis of this work.

## 5. Conclusions

In this work, we identified 558 candidate DEGs and most of them were significantly enriched in protein binding, plasma membrane, extracellular space, and signal transduction. Fifteen module-related DEGs were obtained, and melittin could constrain the expressions of LPAR1, COL5A1, and COL6A2 associated with the PI3K-Akt signaling pathway and CXCL1, CXCL2, and CXCL3 involved in the TNF signal pathway in BC cells. We also found that melittin could induce a significant reduction in proliferation and migration of BC cells. These findings provide a theoretical basis for these six genes as drug-sensitive markers of melittin in BC treatment.

## Figures and Tables

**Table 1:** The primers for qRT-PCR analysis were applied in this work.

Genes	Orientation	Sequences (5′–3′)
LPAR1	Forward	GGCTGCCATCTCTACTTCCATCC
	Reverse	ACAAACAGTGATTCCAAGTCCCA
COL5A1	Forward	ATCTTCGGCTCTCTCAACTCTC
	Reverse	GTCCACATAGGAGAGCAGTTTC
COL6A2	Forward	AGCCTACGGAGAGTGCTACAAG
	Reverse	TGTCCATCGGTCCCGTTCTTGC
CXCL1	Forward	AACCGAAGTCATAGCCACA
	Reverse	TCCTAAGCGATGCTCAAA
CXCL2	Forward	CAAACCGAAGTCATAGCC
	Reverse	GAACAGCCACCAATAAGC
CXCL3	Forward	CAAACCGAAGTCATAGCC
	Reverse	ACCCTCGTAAGAAATAGTCA
GAPDH	Forward	TCCTCACAGTTGCCATGTAGACCC
	Reverse	TGCGGGCTCAATTTATAGAAACCGGG

**Table 2:** The DEGs in GEO data were identified in BC samples.

	Gene names
Upregulated DEGs	MAN1A1, ARHGDIB, PDE1A, SLC1A1, RNF182, CXCL1, ABLIM1, NEFL, GRAMD3, GJA1, TENM2, CFH, BDKRB1, CA8, HAS2, PTCHD1, STC1, NECTIN3, DNER, MARCKS, TRIM6, SYTL2, ARL4C, WNT5A, VCAM1, VEGFC, KLF12, NLGN1, LMO4, TNS3, ATXN1, LRRC15, CLEC2B, HHIP, TMEM45A, DAPK1, EPB41L3, ADGRG6, ENPP2, MLPH, TUBAL3, RUNX2, TMEM200A, EDN1, PAG1, DIXDC1, TCF4, IL1RAPL1, INHBB, ADAM12, EPS8, CNTN1, MYLK, G0S2, EFNA5, TMEM56, MMP2, TBC1D4, SLC16A2, LOXL2, RORA, SLC8A1, GDA, PAQR5, BHLHE41, RCAN3, ADAMTS5, TMEM158, ADAMTS12, DDIT4, DPP7, SNAI2, TENM3, C4orf47, PPARG, DPYSL2, PID1, C15orf48, CXCL3, CLU, FYN, PTGER4, TMPRSS15, CA12, CXCL8, BNIP3L, TFPI2, FBXL7, LRFN5, CHST4 SH3RF3, PDK3, PLPP3, P4HA2, PGM5, TSC22D1, FAM102B, TMEM44, GABBR2, GUCY1A2, NR5A2, MID1, CCL2, AKR1C3, MAP3K7CL, PXDNL, GCNT1, VLDLR, C11orf96, LOC283352, RNASE4, BASP1, PHACTR1, TGFBI, KLF6, HMOX1, ANXA3, SAT1, SLC39A4, ID2, SSBP2, PRUNE2, SLC1A3, AFP, NR2F1-AS1, RGS4, ANG, NDRG1, SLC25A30, NRN1 PFKFB4, ST3GAL5, TSC22D3, PXK, PRKCDBP, MYBL1, TMCO3, CTSB, NREP, PRICKLE2, NKX3–1, PPM1E, MAP1B, CD99, ITGA4, DDR2, SDC2, TUBB2A, CELF2, KANK1, EYA1, CCDC134, ALDH3B1, DPYSL4, IER3, MAP4K4, ATP8B3, LIFR, ARFGEF3, FOSL2, CAV1, NANOS1, KCNN4, PPP1R16A, SAP30, EPAS1, KCTD21, PBX1, TLE4, MICAL3, SIM1, PMEPA1, TAF9B, SMAD6, JUND, PDGFC, FPR1, GYS1, LSS, MXI1, CCDC92, IDH2, RRAS, AAMDC, CXCL2, RIPK4, DPY19L2P2, PEG10, DHRS3, ALDH1A1, MICAL2, CXXC5, DKK3, SELENOM, TCF7L1, IRS1, RFX5, TMSB4X, DCAF6, PRR16 STXBP6, CERK, PTTG1IP, ELL2, LINC00643, LACTB, ZNF385D, LTBP1, CAT, P2RY8 SNAPC3, SYNC, IL31RA, FAM229B, 3\Mar, ATP2B4, RASSF2, DNAJB4, SLC39A10, CUEDC1 LINC00165, MTHFD2L, RIMS3, TXNDC16, C10orf10, NAT1, PLEKHG4, GLCCI1, PIK3R3, LOC286161, NID2, CXADR, TNC, TUBA1A, GSTM4, LOC100506844, OGFRL1, FDFT1, CACNA1A, HERPUD2, CD109, PNRC1, PLIN2, SEC22C, GRK5, DHCR7, RRAGA, KDM4C, DPYSL5, MRGPRX3, CDH2, FAM127B, ANKRD20A5P, FAM127A, IL11RA, PPP4R4, MME, MGST3, LOC101927752, FAM168A, CDYL2, PPP1R3C, ZNF827, CCBE1, MSMO1, TM4SF1, P3H2, NCAM2, YPEL5, PTPRS, INSIG1, CDC42EP3, TCFL5, IL1R1, PRICKLE1, LGALS1, CPEB2, LOC105371967, TRIQK, SCD, FAM134B, LPAR1, GFPT2, COL13A1, FAM133A, F2R, TUSC1, CA9, ENO2, VEGFA, PLAU, KIF3C, KATNAL1, OPTN, FABP4, PDE8A, LOC283140, ADD3, SQLE, COL18A1, INSIG2, TADA3, TMEM170B, ATL1, CCL7, IGSF3, NEURL1B, CHI3L2, COL6A2, CHKB, PAPPA, TGIF1, HTRA1, TBC1D8B, ASAH1, ABCC10, MSC-AS1, VMP1, LOC101927027, IL18, LPCAT2, MYO9A, FLI1, KLF13, HBP1, PRR34-AS1, DHCR24, MGLL, GLCE, CCNG2, TTC13, MOSPD2, LOC100506922, ROBO2, ZNF697, HDAC4, MLF1, CSMD3, CCNA1, SCO2, SLC25A1, BNC2, ZNF704, CD82, PICSAR, FOXP4-AS1, FAM110B, SPAG4, RECK, CLDN1, SH3PXD2A, SNTB1, IRF9, LACC1, EPHX1, WWC3, THAP8, RIMS2, SREBF2, TMOD2, ZNF365, ARHGAP28, COL5A1, IFT74, LOC100130987, LINC01279, SPRY1, PQLC2L, CADM1, C11orf80, MIR210HG, PPP1R13L, KIAA0895, NRP1, PLSCR4, HGSNAT, KRCC1, HIST1H2AC, GPR137C, TNFAIP8, IQGAP3, WIPF1, URB1-AS1, IL17RD, MBD5, REEP6, ANK2, PQLC3, DNAJC6.

Downregulated DEGs	PTPRD, VCAN, HAPLN1, JPH1, DSP, CA2, ZNF804A, ETV1, MTUS1, GULP1, ZNF268, KIT, PLEKHA5, RBP7, VGLL3, AASS, DKK1, KRT8, CHSY3, PTN, SLC7A2, ADRB1, TGFA, HSPB8, ATP7B, PCDH19, SLC18B1, ESM1, MAGEC2, ARHGAP42, ZNF462, SLC16A7, TMEM5, SOCS2, SYBU, MFAP5, GPC4, KHDRBS3, CHMP4C, AMPH, ST8SIA4, RGS2, LINC01234, SLAIN1, KCNQ3, TMEM246, ST3GAL1, GHR, ZNF711, LAMA1, PLEKHG3, ITPR2, ZNF518B, DOCK4, WISP3, SERPINE2, NFXL1, PLCXD2, ZNF350, MLKL, DLL1, ZNF331, DMKN, FAM171B, RPS6KA6, PDGFRL, KIAA1551, ZDHHC15, MYH2, NCBP3, BRAP, SOX7, PELP1, IL4R, HPD, STAC, METTL7B, IFI44, APBB2, SPOUT1, ENDOD1, HBEGF, MPP5, ESF1, MCM9, JAG1, SERPINB7, CYB5R2, MATR3, ANO5, CTNS, MOB3B, GFRA1, CCDC181, KIAA1671, EBI3, BEX5, KBTBD8, CD163L1, CASP4, LOC729732, RRAGD, CPNE8, ANKRD1, ITGA2, STK38L, TSR1, DGKI, TEX10, SWI5, HRK, TOX2, ZNF547, ZNF615, LOC400043, INTS6, CKB, GBP3, CCDC3, PRKAR2B, MBOAT1, ZNF708, RBBP6, SH3GL3, IL13RA1, PSMB8, ITGBL1, CEP126, RAP1GAP2, GLT8D2, ZNF605, CEP290, IL15RA, ZNF107, ZNF286A, PREX1, RNF125, SP110, ZNF267, PKP2, C11orf70, LIMCH1, NAV3, MBNL3, AKAP9, TMEM156, EHD1, MAP3K14, TBC1D30, CCDC85C, CHST2, LMO2, INPP4B, TMEM133, ERVMER61–1, ZNF91, CDK9, ZNF492, HMGN5, SEMA3A, MANSC1, IGFBP2, ELOVL4, UPP1, RNF175, ASAP1-IT1, LY6K, SLC2A13, LOC100506691.

**Table 3:** Top thirty enriched GO terms of DEGs in BC according to *P* value.

Term	Description	Count	Genes
GO:0005615	Extracellular space (CC)	67	NRP1, IL18, EDN1, CXADR, MMP2, ASAH1, CKB, ESF1, GPC4, SERPINE2, WISP3, ANG, PAPPA, HTRA1, IL4R, HMOX1, CCBE1, TGFBI, CFH, TGFA, IL15RA, PDGFC, CAT, SEMA3A, LOXL2, EBI3, MTUS1, GHR, INHBB, VEGFC, VEGFA, SERPINB7, STC1, VCAN, CTSB, CA2, ADAMTS5, CXCL1, WNT5A, CCL2, ENPP2, TNC, CXCL3, CLU, CXCL2, CD109, CXCL8, KIT, NRN1, CCL7, VCAM1, ENO2, COL6A2, PTN, GCNT1, COL18A1, LGALS1, NLGN1, CHI3L2, PXDNL, DKK3, LAMA1, AFP, DKK1, HBEGF, IGFBP2, PLAU
GO:0007157	Heterophilic cell-cell adhesion via plasma membrane cell (BP)	10	VCAM1, PTPRD, CADM1, TENM2, TENM3, NLGN1, NECTIN3, CDH2, CXADR, IL1RAPL1
GO:0071356	Cellular response to tumor necrosis factor (BP)	14	VCAM1, PID1, HDAC4, CCL2, EDN1, CXCL8, NKX3-1, FABP4, HAS2, RORA, ANKRD1, ADAMTS12, ZNF268, CCL7
GO:0071456	Cellular response to hypoxia (BP)	13	SLC8A1, EPAS1, CPEB2, EDN1, RORA, ANKRD1, HMOX1, VEGFA, BNIP3L, PTN, NKX3-1, NDRG1, STC1
GO:0004896	Cytokine receptor activity (MF)	8	IL4R, LIFR, IL15RA, IL13RA1, IL11RA, EBI3, GHR, IL31RA
GO:0050839	Cell adhesion molecule binding (MF)	10	VCAM1, PTPRD, CADM1, TENM2, TENM3, NLGN1, DSP, NECTIN3, ITGA4, CXADR
GO:0006695	Cholesterol biosynthetic process (BP)	8	MSMO1, INSIG2, SQLE, DHCR7, INSIG1, LSS, FDFT1, DHCR24
GO:0001666	Response to hypoxia (BP)	16	CAV1, CCL2, EPAS1, ITGA2, MMP2, DDIT4, ITPR2, VCAM1, VEGFC, CA9, ANG, HMOX1, VEGFA, CAT, LOXL2, PLAU
GO:0030198	Extracellular matrix organization (BP)	17	RECK, COL18A1, HAPLN1, COL13A1, TNC, ITGA2, ITGA4, NID2, DDR2, COL5A1, VCAM1, LAMA1, TGFBI, COL6A2, VCAN, APBB2, MFAP5
GO:0014704	Intercalated disc (CC)	8	SLC8A1, PGM5, ANK2, PKP2, GJA1, DSP, CDH2, CXADR
GO:0001525	Angiogenesis (BP)	18	SAT1, COL18A1, CAV1, NRP1, CCL2, EPAS1, IL18, CXCL8, RORA, JAG1, ESM1, MMP2, VEGFC, ANG, HMOX1, VEGFA, TGFBI, TGFA
GO:0007155	Cell adhesion (BP)	28	CCL2, TNC, CDH2, DDR2, ITGBL1, VCAM1, WISP3, TGFBI, COL6A2, ROBO2, LOXL2, COL18A1, HAPLN1, NLGN1, CD99, ITGA2, PTPRS, CHST4, NECTIN3, ITGA4, NID2, COL5A1, LAMA1, NCAM2, PGM5, CNTN1, VCAN, ADAM12
GO:0005604	Basement membrane (CC)	10	P3H2, COL18A1, LAMA1, TNC, TGFBI, PTN, EFNA5, NID2, LOXL2, COL5A1
GO:0060326	Cell chemotaxis (BP)	9	CXCL1, VCAM1, CCL2, CXCL2, FPR1, HBEGF, LPAR1, KIT, DOCK4
GO:0030165	PDZ domain binding (MF)	10	ADRB1, ATP2B4, CADM1, SNTB1, NLGN1, GJA1, LPAR1, CXADR, SDC2, DOCK4
GO:0008284	Positive regulation of cell proliferation (BP)	27	TNC, EDN1, ESM1, KIT, HMGN5, IL31RA, AKR1C3, TGFA, PTN, NKX3-1, PDGFC, ZNF268, RUNX2, COL18A1, LIFR, DLL1, IRS1, TNS3, VEGFC, HDAC4, VEGFA, KDM4C, HBEGF, PBX1, HAS2, GRK5, F2R
GO:0071347	Cellular response to interleukin-1 (BP)	9	CCL2, EDN1, CXCL8, NKX3-1, HAS2, RORA, ANKRD1, ADAMTS12, CCL7
GO:0003779	Actin binding (MF)	19	ABLIM1, DIXDC1, PHACTR1, MLPH, MICAL2, MYH2, MICAL3, PXK, MYO9A, EPB41L3, EPS8, ANG, LIMCH1, SNTB1, TMOD2, WIPF1, ADD3, STK38L, MYLK
GO:0005886	Plasma membrane (CC)	145	CADM1, JAG1, LPAR1, MMP2, KANK1, IL31RA, PRKAR2B, ATP2B4, ANK2, HTRA1, TGFBI, IL15RA, RRAS, PDGFC, MLKL, PAG1, MTUS1, EBI3, RECK, CA12, LIFR, PTPRS, ABCC10, DLL1, BASP1, IRS1, IL11RA, NCAM2, CD163L1, IGSF3, DSP, EFNA5, ADD3, WNT5A, SLC2A13, IL1R1, CAV1, ENPP2, CSMD3, MME, BDKRB1, PXK, NRN1, PAQR5, P2RY8, PPP1R16A, NDRG1, TBC1D30, RAP1GAP2, LRFN5, CDC42EP3, IL1RAPL1, MSMO1, ADGRG6, ITGA2, ITGA4, PCDH19, DOCK4, RGS2, SLC7A2, PKP2, RGS4, SYTL2, MARCKS, GRK5, DNAJB4, PLAU, CACNA1A, NRP1, PREX1, GJA1, GABBR2, CXADR, DDR2, SDC2, GPC4, SPRY1, SLC1A3, KCNQ3, HMOX1, GUCY1A2, TGFA, MGLL, ANO5, CAT, IL13RA1, JPH1, SLC1A1, ATP8B3, GHR, PTGER4, MICAL3, MPP5, NECTIN3, ALDH3B1, DAPK1, EPB41L3, ADRB1, CA9, STXBP6, CD82, CNTN1, CA2, ADAM12, ARL4C, PMEPA1, PTCHD1, SLC39A10, FPR1, CD109, CDH2, KIT, PLPP3, VCAM1, PLIN2, CLEC2B, DNER, ENO2, CERK, VMP1, EHD1, SLC39A4, FAM127A, PTPRD, SLC8A1, COL13A1, MAP1B, NLGN1, LY6K, CD99, DGKI, ANXA3, ITPR2, KCNN4, DKK1, PLSCR4, SLC16A7, FYN, BNC2, TENM2, GFRA1, HBEGF, CTNS, F2R, VLDLR
GO:0009986	Cell surface (CC)	29	WNT5A, IL1R1, NRP1, CLU, CD109, LPAR1, CXADR, SDC2, VCAM1, KCNQ3, SLC1A3, TGFA, PTN, ROBO2, PDGFC, HHIP, LRFN5, IL1RAPL1, GHR, LGALS1, NLGN1, ITGA2, LY6K, ITGA4, VEGFA, IGSF3, HBEGF, PLAU, F2R
GO:0019221	Cytokine-mediated signaling pathway (BP)	12	CCL2, SOCS2, LIFR, IL15RA, KIT, LRRC15, IL13RA1, IL17RD, IL11RA, EBI3, GHR, IL31RA
GO:0007165	Signal transduction (BP)	52	NRP1, PPARG, IQGAP3, GJA1, IL17RD, DDR2, ANK2, STAC, WISP3, IL4R, INTS6, GUCY1A2, IL15RA, PDE8A, PAG1, SH3GL3, ARHGAP28, OPTN, IRS1, DAPK1, VEGFC, INPP4B, CXCL1, CCL2, GULP1, FPR1, CXCL8, AKAP9, KIT, SP110, CXXC5, MYO9A, CCL7, PDE1A, ARHGAP42, CDC42EP3, IL1RAPL1, EPAS1, LGALS1, DPYSL5, DPYSL2, ITPR2, MGST3, RPS6KA6, EPS8, TENM2, TENM3, HBEGF, IGFBP2, PLCXD2, PLAU, VLDLR
GO:0009612	Response to mechanical stimulus (BP)	8	INHBB, CCL2, PTGER4, TNC, JUND, PPARG, BDKRB1, IGFBP2
GO:0005578	Proteinaceous extracellular matrix (CC)	18	WNT5A, COL18A1, HAPLN1, LTBP1, LGALS1, MMP2, COL5A1, GPC4, LAMA1, WISP3, CCBE1, VEGFA, TGFBI, COL6A2, VCAN, ADAMTS12, TFPI2, ADAMTS5
GO:0030426	Growth cone (CC)	11	NRP1, EPS8, ANG, PREX1, TENM2, TMOD2, DPYSL2, BASP1, APBB2, CXADR, NEFL
GO:0005518	Collagen binding (MF)	8	TGFBI, CCBE1, ADGRG6, ITGA2, NID2, CTSB, LRRC15, DDR2
GO:0007399	Nervous system development (BP)	19	CXCL1, GDA, MAP1B, DPYSL5, NLGN1, MBD5, DPYSL4, JAG1, DPYSL2, NRN1, HDAC4, ST8SIA4, VEGFA, TMOD2, PTN, GFRA1, EFNA5, SIM1, VLDLR
GO:0005515	Protein binding (MF)	282	PRR16, LTBP1, CADM1, TUBB2A, EDN1, RORA, LPAR1, MXI1, MAGEC2, ATP2B4, FLI1, SERPINE2, STAC, INSIG2, CCBE1, INSIG1, INTS6, CFH, CEP290, CCNA1, SH3GL3, RECK, MYH2, LIFR, PTPRS, PRKCDBP, OPTN, BRAP, VEGFC, SPAG4, AAMDC, VEGFA, TGIF1, ADAMTS5, RBP7, IL1R1, TADA3, CCDC92, TAF9B, MME, BDKRB1, RIMS2, MYO9A, PPP1R3C, SYBU, GYS1, TCF4, RAP1GAP2, IL1RAPL1, GCNT1, KLF6, KLF12, KLF13, LGALS1, SMAD6, LAMA1, EPS8, RGS2, PKP2, SYTL2, GRK5, PLAU, NRP1, PPARG, GJA1, RNF182, CKB, FAM168A, SWI5, ZNF350, DMKN, IL4R, TGFA, NEURL1B, HHIP, IL13RA1, BHLHE41, GHR, SH3PXD2A, SNAPC3, TLE4, NECTIN3, RNF175, RBBP6, INHBB, EPB41L3, VCAN, TMSB4X, G0S2, MAP3K14, ARL4C, THAP8, CEP126, CXCL2, FPR1, CXCL8, ZNF365, KIT, MLF1, AMPH, PLEKHG4, COL6A2, ETV1, HBP1, VMP1, WIPF1, CERK, FAM127B, SLC8A1, PDK3, DGKI, NID2, MID1, SNAI2, AFP, DKK1, PLSCR4, FYN, BNIP3L, RASSF2, GFPT2, SH3RF3, PBX1, APBB2, KATNAL1, NR5A2, TEX10, VLDLR, LMO2, CHMP4C, IL18, LMO4, ANKRD1, JAG1, MMP2, KANK1, PRKAR2B, GSTM4, ANK2, ELOVL4, TGFBI, RRAS, IL15RA, ROBO2, MLKL, PDGFC, EBI3, PAG1, NCBP3, TMPRSS15, PID1, SOCS2, HRK, DLL1, BASP1, URB1-AS1, IRS1, TNS3, MAP4K4, HSPB8, TNFAIP8, DSP, RIPK4, GBP3, WNT5A, CAV1, CLU, RRAGA, SOX7, CXXC5, RRAGD, CCL7, PAQR5, PEG10, PELP1, PPP1R16A, CDYL2, JUND, NKX3-1, NDRG1, MBNL3, RUNX2, STK38L, ARHGDIB, REEP6, DIXDC1, EPAS1, RFX5, ITGA2, ITGA4, SELENOM, PSMB8, SREBF2, DOCK4, HDAC4, NREP, PLEKHA5, PRICKLE1, TXNDC16, SLAIN1, PNRC1, BEX5, PTTG1IP, DNAJB4, MYLK, CACNA1A, ATP7B, SAT1, IER3, FOSL2, CYB5R2, MLPH, ATL1, PREX1, GABBR2, CXADR, DDR2, SDC2, SAP30, FAM133A, SPRY1, ANG, HMOX1, TRIM6, SNTB1, MOB3B, TUBA1A, LOXL2, SLC1A1, KHDRBS3, PTGER4, MPP5, CDK9, ALDH3B1, DAPK1, FAM134B, EYA1, ADRB1, PPM1E, CA8, CD82, ZNF711, CLDN1, INPP4B, CTSB, CA2, MATR3, PMEPA1, ABLIM1, PPP4R4, NANOS1, AKAP9, CDH2, GRAMD3, MAP3K7CL, PLPP3, TSC22D1, DNER, KRT8, LACC1, ENO2, SEC22C, ADAMTS12, PIK3R3, EHD1, NEFL, SCO2, PTPRD, COL13A1, MAP1B, DPYSL5, DPYSL4, DPYSL2, RCAN3, PPP1R13L, MGST3, IRF9, ATXN1, KCNN4, ID2, IGFBP2, F2R
GO:0031012	Extracellular matrix (CC)	19	COL18A1, HAPLN1, LTBP1, LGALS1, TNC, CLU, NID2, MMP2, COL5A1, LAMA1, SERPINE2, HTRA1, TGFBI, COL6A2, DSP, VCAN, ADAMTS12, LOXL2, TFPI2
GO:0008201	Heparin binding (MF)	13	CCL2, NRP1, COL13A1, CCL7, COL5A1, SERPINE2, ANG, WISP3, VEGFA, CFH, PTN, HBEGF, ADAMTS5

**Table 4:** Top fifteen significant signaling pathways associated with DEGs in BC according to *P* value.

ID	Term	Count	*P* value	Genes
hsa00100	Steroid biosynthesis	6	0.000342	MSMO1, **SQLE**, DHCR7, **LSS**, **FDFT1**, DHCR24
hsa04514	Cell adhesion molecules (CAMs)	12	0.005828	VCAM1, NCAM2, CADM1, CLDN1, NLGN1, CNTN1, CD99, VCAN, NECTIN3, ITGA4, CDH2, SDC2
hsa04540	Gap junction	9	0.007066	ADRB1, TUBB2A, GUCY1A2, TUBAL3, GJA1, PDGFC, **LPAR1**, **TUBA1A**, ITPR2
hsa04060	Cytokine-cytokine receptor interaction	15	0.015894	IL1R1, CCL2, IL18, LIFR, CXCL8, KIT, IL11RA, CCL7, VEGFC, IL4R, VEGFA, IL15RA, PDGFC, IL13RA1, GHR
hsa00910	Nitrogen metabolism	4	0.015944	CA9, CA8, CA12, CA2
hsa05202	Transcriptional misregulation in cancer	12	0.019138	EYA1, FLI1, ID2, LMO2, PPARG, CXCL8, **CDK9**, ETV1, PBX1, RUNX2, PLAU, MLF1
hsa04668	TNF signaling pathway	9	0.020338	**CXCL1**, VCAM1, CCL2, **CXCL3**, EDN1, **CXCL2**, MLKL, MAP3K14, PIK3R3
hsa04974	Protein digestion and absorption	8	0.022588	**COL18A1**, KCNN4, SLC8A1, **COL13A1**, **COL6A2**, MME, SLC1A1, **COL5A1**
hsa05412	Arrhythmogenic right ventricular cardiomyopathy	7	0.025957	PKP2, GJA1, DSP, ITGA2, ITGA4, CDH2, TCF7L1
hsa04151	PI3K-Akt signaling pathway	19	0.028358	TNC, ITGA2, **LPAR1**, KIT, ITGA4, IRS1, **COL5A1**, DDIT4, LAMA1, VEGFC, IL4R, VEGFA, GYS1, **COL6A2**, EFNA5, PDGFC, PIK3R3, GHR, F2R
hsa04510	Focal adhesion	13	0.033173	CAV1, TNC, ITGA2, ITGA4, **COL5A1**, VEGFC, LAMA1, **FYN**, VEGFA, **COL6A2**, PDGFC, PIK3R3, MYLK
hsa04150	mTOR signaling pathway	6	0.037778	RPS6KA6, RRAGA, RRAGD, PIK3R3, IRS1, DDIT4
hsa05219	Bladder cancer	5	0.041527	VEGFA, CXCL8, HBEGF, MMP2, DAPK1
hsa05200	Pathways in cancer	20	0.047909	WNT5A, EPAS1, PTGER4, PPARG, CXCL8, ITGA2, **BDKRB1**, **LPAR1**, KIT, MMP2, TCF7L1, DAPK1, LAMA1, VEGFC, VEGFA, TGFA, NKX3-1, HHIP, PIK3R3, F2R
hsa04630	Jak-STAT signaling pathway	9	0.048544	IRF9, SOCS2, IL4R, LIFR, IL15RA, PIK3R3, IL13RA1, IL11RA, GHR

The bold represented the key genes in the modules.

**Table 5:** Twelve module networks analyzed by MCODE.

Cluster	Score	Nodes	Edges	Node IDs
1	8	8	28	**CXCL2**, **CXCL1**, IL8, GABBR2, FPR1, **BDKRB1**, **LPAR1**, **CXCL3**
2	6	6	15	SEMA3A, DPYSL4, DPYSL5, DPYSL2, **FYN**, NRP1
3	5.6	6	14	**COL6A2**, P4HA2, **COL18A1**, **COL13A1**, **COL5A1**, LEPREL1
4	5	5	10	**TUBA1A**, OPTN, AKAP9, CEP290, PRKAR2B
5	4	4	6	**FDFT1**, SREBF2, **SQLE**, **LSS**
6	4	4	6	SNAPC3, INTS6, **CDK9**, ELL2
7	4	4	6	FBXL7, RBBP6, RNF182, KBTBD8
8	3.5	5	7	TRIM6, IRF9, MID1, TRIM2, GBP3
9	3.143	8	11	WNT5A, SH3GL3, KIT, CLU, VEGFC, TMSB4X, DNAJC6, AMPH
10	3	3	3	TUBB2A, TUBAL3, GJA1
11	3	3	3	NEURL1B, DLL1, JAG1
12	3	3	3	GSTM4, MGST3, EPHX1

The bold represented the key genes in the modules.

## Abbreviations

BC: Bladder cancer
GEO: Gene Expression Omnibus
DEGs: Differentially expressed genes
GO: Gene ontology
KEGG: Kyoto Encyclopedia of Genes and Genomes
PPI: Protein–protein interaction
DAVID: Database for Annotation, Visualization, and Integrated Discovery
STRING: Search Tool for the Retrieval of Interacting Genes
MCODE: Molecular Complex Detection
FBS: Fetal bovine serum
BP: Biological process
MF: Molecular function
CC: Cellular component.

## Supplementary Materials

Supplementary Materials The PPI network was clearly shown in the Supplementary Material for zooming in and out as needed. The larger the node diameter is, the smaller the *P* value and the more significant the node is.
