# Supplementary figures and images for: Melittin Constrains the Expression of Identified Key Genes Associated with Bladder Cancer
Source: J Immunol Res. 2018 May 3;2018:5038172. doi: 10.1155/2018/5038172 (PMC5960535; doi:10.1155/2018/5038172)

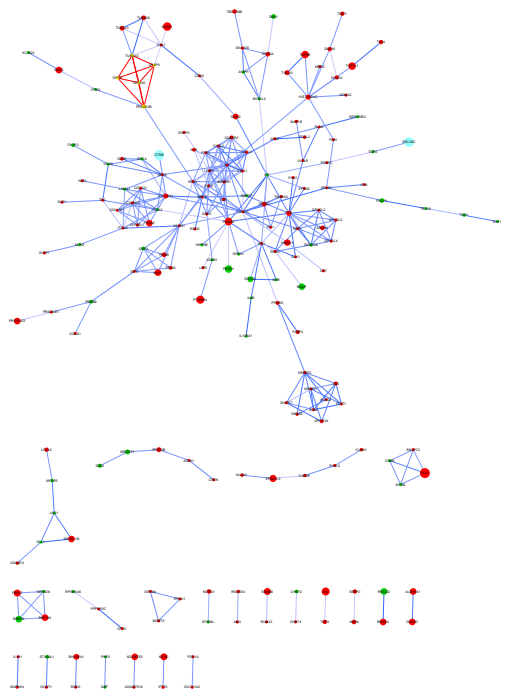

Supplement: Supplementary Materials — The PPI network was clearly shown in the Supplementary Material for zooming in and out as needed. The larger the node diameter is, the smaller the P value and the more significant the node is. [file 5038172.f1.pdf]
